# Analysis of Venezuelan Equine Encephalitis Replicon Particles Packaged in Different Coats

**DOI:** 10.1371/journal.pone.0002709

**Published:** 2008-07-16

**Authors:** Kurt I. Kamrud, Kim D. Alterson, Chasity Andrews, Laura O. Copp, Whitney C. Lewis, Bolyn Hubby, Deepa Patel, Jonathan O. Rayner, Todd Talarico, Jonathan F. Smith

**Affiliations:** 1 AlphaVax, Inc., Research Triangle Park, North Carolina, United States of America; 2 Liquidia Technologies, Inc., Research Triangle Park, North Carolina, United States of America; 3 Midwest Research Institute, Kansas City, Missouri, United States of America; Yale University, United States of America

## Abstract

**Background:**

The Venezuelan equine encephalitis (VEE) virus replicon system was used to produce virus-like replicon particles (VRP) packaged with a number of different VEE-derived glycoprotein (GP) coats. The GP coat is believed to be responsible for the cellular tropism noted for VRP and it is possible that different VEE GP coats may have different affinities for cells. We examined VRP packaged in four different VEE GP coats for their ability to infect cells *in vitro* and to induce both humoral and cellular immune responses *in vivo*.

**Methodology/Principal Findings:**

The VRP preparations were characterized to determine both infectious units (IU) and genome equivalents (GE) prior to *in vivo* analysis. VRP packaged with different VEE GP coats demonstrated widely varying GE/IU ratios based on Vero cell infectivity. BALB/c mice were immunized with the different VRP based on equal GE titers and the humoral and cellular responses to the expressed HIV *gag* gene measured. The magnitude of the immune responses measured in mice revealed small but significant differences between different GP coats when immunization was based on GE titers.

**Conclusions/Significance:**

We suggest that care should be taken when alternative coat proteins are used to package vector-based systems as the titers determined by cell culture infection may not represent accurate particle numbers and in turn may not accurately represent actual *in vivo* dose.

## Introduction

Venezuelan equine encephalitis (VEE) virus is a member of the *Togaviridae* family within the *Alphavirus* genus. Alphaviruses have single-stranded, positive-sense, RNA genomes that are capped at the 5′ end and polyadenylated at the 3′ end. The viral nonstructural proteins are encoded for in the 5′ two-thirds of the genome and the structural proteins are encoded in the 3′ one-third of the genome. The nonstructural proteins are translated in cells directly from the capped input RNA, whereas the structural proteins are translated from a subgenomic RNA transcribed from a 26S promoter present on the full-length, negative-stranded, RNA replication intermediate (reviewed in [1]). A number of live attenuated VEE virus variants have been described [2]–[7]. One of these attenuated VEE viruses (V3014) was used to generate a replicon system that has been used as a vaccine vector to express a wide array of genes [8], [9]. Such recombinant replicons are generated by replacing the structural protein coding region with genes of interest (GOI) generating what is essentially a self-replicating mRNA. Because the replicon RNA does not contain the structural genes for VEE, it is a single-cycle, propagation-defective RNA and replicates only within the cell into which it is introduced. The replicon RNA can be packaged into virus-like replicon particles (VRP) by supplying the structural protein genes of VEE *in trans* [8].

VEE virus and VRP derived from the VEE replicon system have a demonstrated lymphotropism [1], [10]–[12]. This characteristic may, in part, explain the robust antigen-specific immune responses, both humoral and cellular, detected in animals immunized with VRP vaccines [8], [13]–[26]. The cell tropism of VEE virus (or VRP) is believed to be defined by the envelope proteins that are embedded in the membrane that forms the outer surface of the particles. Amino acid changes in the envelope glycoproteins (GP) of VEE viruses have been shown to affect the relative rate of virus penetration of cells in culture [2], relative heparan sulfate or hydroxylapatite column binding [7], [27], *in-vivo* serum virus clearance rates and overall virus attenuation in animal models [5], [7], [27]–[29]. In addition, single or multiple amino acid changes in the GP coat have been shown to delay VEE virus progression to, or spread beyond, the draining lymph node of footpad-inoculated mice, while wild type VEE virus progresses on into the serum and seeds multiple other organs [5], [30]. It is assumed that VRP packaged with mutant GP coats would maintain the same surface properties and cell tropisms of the parent VEE virus harboring the same GP mutation(s) [10]. Considering this, VRP packaged with mutant GP coats, which in the context of live VEE viruses demonstrate altered phenotypes, tropisms and different trafficking to and from draining lymph nodes, may be less immunogenic than VRP packaged with the wild type (V3000) VEE GP coat.

Previous experience using VRP packaged with two different VEE GP coats, V3000 (the wild type Trinidad donkey GP coat) and V3014, has suggested that different GP coats can induce different levels of immunogenicity in mice (Kamrud and Smith, unpublished data) possibly due to lower dendritic cell tropism of the V3014 GP coat [10]. We were interested in determining the effects of different GP coats, derived from several live VEE viruses, on the immunogenicity of single-cycle VRP in BALB/c mice. Here we report the relative immunogenicity (both cellular and humoral) *in vivo* of VRP packaged with GP coats from wild-type and three attenuated variants of VEE virus in BALB/c mice and additionally demonstrate that the GP coats impart variable *in vitro* Vero cell infectivity relative to one another.

## Results

### Quantitative reverse transcription PCR (RTqPCR) analysis of VRP genome copies

A replicon expressing the HIV *gag* gene was packaged with each the following VEE GP coats: V3014 GP, V3000 GP, V3042 GP and TC-83 GP. The GP genes were identified by the name of the VEE infectious clones from which they were derived; V3000, V3014, V3042 and TC-83. A list of the specific mutations found in each of the glycoprotein coats is shown in Table 1. Infectious titers were determined for each preparation of VRP on Vero cells and are referred to as infectious units (IU). The number of replicon genome equivalents (GE) was also determined for each VRP preparation by quantitative reverse transcription PCR (RTqPCR ) using primers specific for the nsP2 gene region. Determination of the number of GE in a VRP preparation is an estimate of the total number of physical particles. A summary of the IU and GE titers is shown in Table 2. VRP packaged with the V3000 and V3042 GP coats demonstrated much higher GE/IU ratios than VRP packaged with V3014 or TC-83 GP coats (Table 2). These data suggest that a large number of particles packaged with the V3000 and V3042 GP coats are not detected in a normal infectivity assay carried out in Vero cells.

**Table 1:** Structural protein coding region and location of glycoprotein mutations in different VEE viruses

VEE virus infectious clone	VEE glycoprotein (amino acid number)						Relative heparan sulfate binding
	E2 (120)	E2 (209)	E2 (239)	E2 (323)	E1 (81)	E1 (272)	
V3000	T	E	I	G	F	A	Weak
V3014	T	K	N	G	F	T	Strong
TC-83	R	E	N	E	F	A	Strong
V3042	T	E	I	G	I	A	Weak

**Table 2:** Comparison of genome equivalents

VEE GP coat	IU Titer^a^	GE Titer^b^	GE/IU
V3014	4.90×10^9^	2.22×10^11^	45
TC-83	3.00×10^10^	6.92×10^11^	23
V3000	3.90×10^8^	1.40×10^12^	3590
V3042	5.30×10^8^	6.60×10^11^	1245

a Infectious unit (IU) titer determined on Vero cells. IU/ml titer represented.

b Genome equivalent (GE) titer determined by quantitative reverse transcription PCR. GE/ml titer represented.

### Effect of NaCl concentration on Vero cell VRP infectivity

The V3014 and TC-83 GP coats contain heparan binding mutations at E2-209 and E2-120, respectively [8], [31]. To determine whether the high GE/IU ratios for V3000 and V3042 GP packaged VRP were due to different Vero cell binding affinities, alternative buffers were used during the cell adsorption step of the titration assay. Both the pH and NaCl concentration of the titration buffers were altered from what is normally found in the MEM medium (pH 7.4, 150 mM NaCl) used for sample dilution. Reducing the concentration of NaCl also changed the osmolality of the titration buffers so sucrose was added in place of NaCl to maintain the osmolality normally found in growth medium (∼270 mOsm). Results of the titration assays are summarized in Table 3. Reducing the NaCl concentration (from 150 mM to ≤3.25 mM) in the titration buffer resulted in a dramatic increase in IU titer for both the V3000 and V3042 GP packaged VRP. The majority of these particles therefore contain fully functional replicon RNAs but are differentially infectious *in vitro*. Little change was noted in apparent VRP titer between the different pH levels tested, suggesting that the NaCl concentration of the titration buffer was the most important variable responsible for this effect. A result of this increase in VRP titers was that the GE/IU ratios for V3000 and V3042 GP packaged VRP dropped 58 to 38 fold, respectively (Table 3). However, even with the more sensitive infectivity assay the GE/IU ratios still remained 5 to 10 times higher than the ratios for V3014 and TC-83 GP packaged VRP. The low NaCl concentration buffers did not increase the IU titers of V3014 or TC-83 GP packaged VRP (data not shown).

**Table 3:** Effect of pH and NaCl on Vero cell VRP titer

VEE GP coat	Titration buffer (pH)	IU/ml^c^	GE^d^/IU
V3000	Media (7.4)	1.5×10^8^	9333
V3000	Phosphate^a^ (7.0)	6.3×10^9^	222
	Phosphate (7.4)	8.7×10^9^	160
V3000	Tris^b^ (7.4)	4.2×10^9^	333
V3000	Tris (8.0)	4.0×10^9^	350
V3042	Media (7.4)	5.7×10^7^	11578
V3042	Phosphate (7.0)	2.1×10^9^	314
V3042	Phosphate (7.4)	1.8×10^9^	366
V3042	Tris (7.4)	2.2×10^9^	300
V3042	Tris (8.0)	1.2×10^9^	550

a Phosphate: 20 mM sodium phosphate buffer + 5.5% sucrose at designated pH used to titrate VRP packaged with indicated VEE GP coat.

b Tris: 20 mM trishydroxymethylaminomethane + 5.5% sucrose at designated pH used to titrate VRP packaged with indicated VEE GP coat.

c Infectious unit (IU) titer determined on Vero cells.

d Genome equivalent (GE) titer determined by quantitative reverse transcription PCR.

### Effect of GP coat on VRP immunogenicity

**Figure 1:**
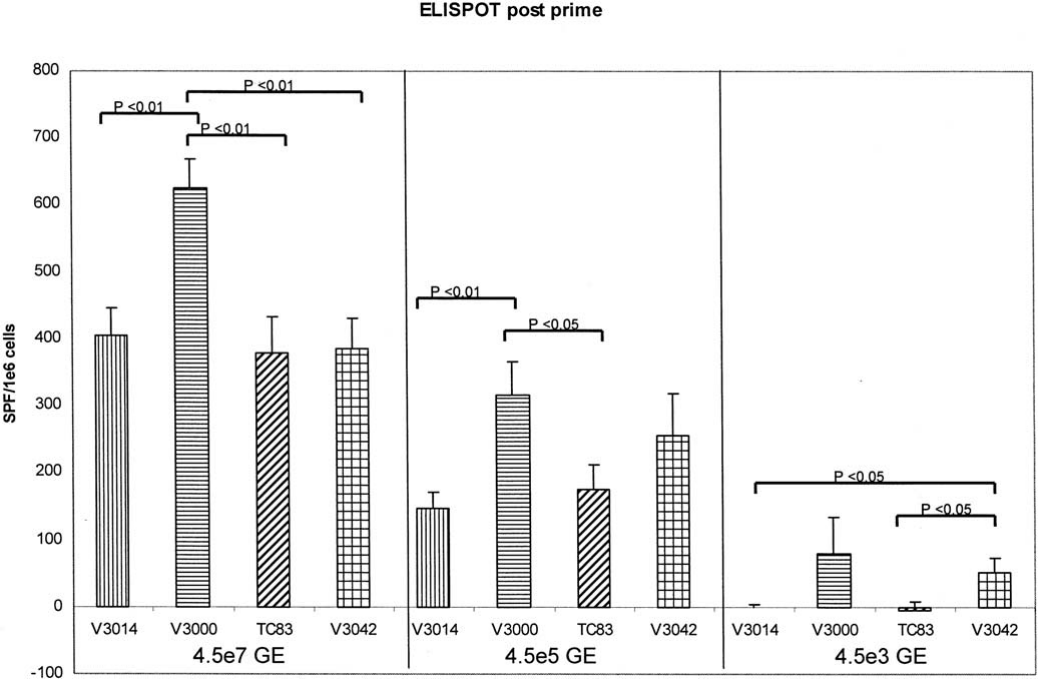
GAG-specific ELISPOT analysis post prime. Splenocytes were isolated from individual animals and GAG specific gamma interferon ELISPOT assays were performed to determine the number of antigen-specific cytokine-secreting T cells. Error bars represent 1 standard error.

**Figure 2:**
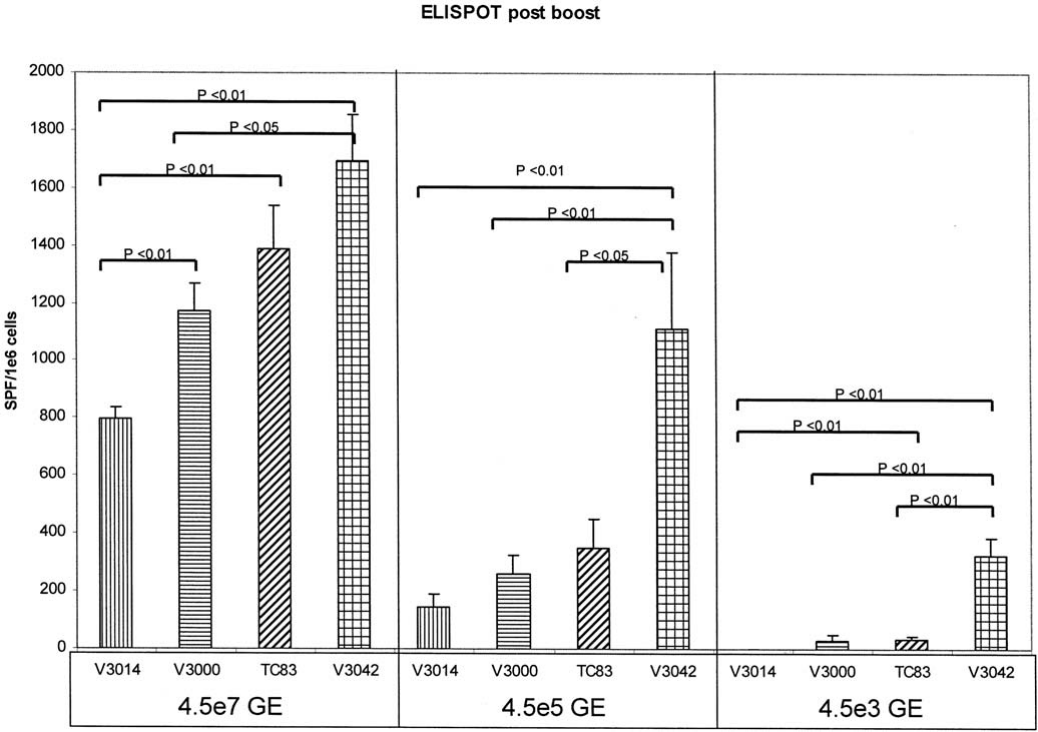
GAG-specific ELISPOT analysis post boost. Splenocytes were isolated from individual animals and GAG specific gamma interferon ELISPOT assays were performed to determine the number of antigen-specific cytokine-secreting T cells. Error bars represent 1 standard error.

**Figure 3:**
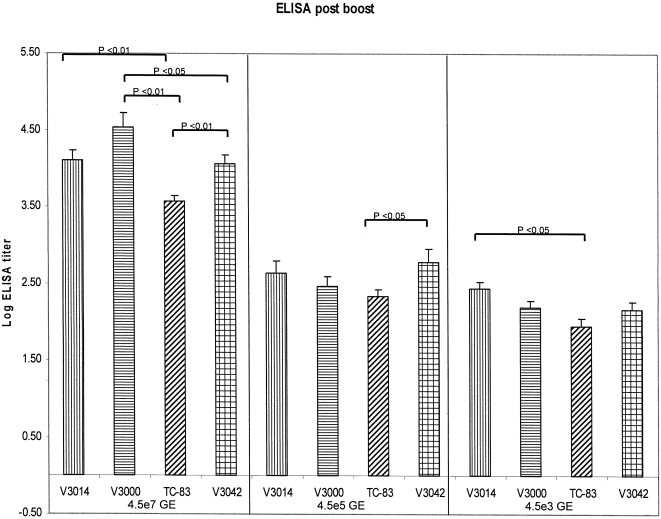
GAG-specific ELISA analysis post boost. Sera was collected from individual animals 1 week after the booster vaccination and GAG-specific ELISA analysis was performed. Error bars represent 1 standard error.

Because VRP IU titer determined on Vero cells detected only a minority of the particles packaged with the V3000 or V3042 GP coats, quantitative RT- PCR was used to determine the relative GE titer for each preparation. In order to detect possible differences in immunogenicity imparted to VRP packaged with different VEE GP coats, a range of GE-based titers were used to immunize mice (Table 4). All of the VRP used in experiments (*in vitro* and *in vivo*) were purified using the same purification protocol. Sixteen mice were immunized at three week intervals with each of three different GE doses. Sera were collected one week after each immunization. Eight mice per group were sacrificed for T-cell analysis 7 days after the prime and the remaining animals were sacrificed 7 days after the boost. The results of GAG-specific ELISPOT and ELISA analysis are summarized in Figures 1, 2, 3. There was a dose dependent, antigen-specific, ELISPOT response detected 7 days after the priming immunization (Figure 1). Animals immunized with the V3000 GP packaged VRP showed higher numbers of spot forming cells (SFC) then animals immunized with VRP packaged with any of the other GP coats at the 4.5×10^7^ GE dose tested. A similar result was noted at the 4.5×10^5^ GE dose, with the exception of the V3042 GP packaged VRP which were not significantly different from V3000 GP packaged VRP (Figure 1). Differences were also noted at the lowest dose tested (4.5×10^3^ GE), the V3042 GP packaged VRP demonstrated significantly more SFCs than V3014 or TC-83 GP packaged VRP (Figure 1). ELISA analysis of serum collected at this time point revealed that titers above background could not be detected (data not shown).

**Table 4:** VEE GP coat mouse immunogenicity study design

Group	VEE GP coat	GE dose^a^	IU dose^b^	# animals
1	V3014	4.5×10^7^	1.00×10^6^	16
2	V3000	4.5×10^7^	1.20×10^4^	16
3	TC-83	4.5×10^7^	1.90×10^6^	16
4	V3042	4.5×10^7^	3.60×10^4^	16
5	V3014	4.5×10^5^	1.00×10^4^	16
6	V3000	4.5×10^5^	1.20×10^2^	16
7	TC-83	4.5×10^5^	1.90×10^4^	16
8	V3042	4.5×10^5^	3.60×10^2^	16
9	V3014	4.5×10^3^	1.00×10^2^	16
10	V3000	4.5×10^3^	1.2	16
11	TC-83	4.5×10^3^	1.90×10^2^	16
12	V3042	4.5×10^3^	3.6	16

a Genome equivalent (GE) titer determined by quantitative reverse transcription PCR.

b Infectious unit (IU) titer determined on Vero cells.

ELISPOT analysis of samples collected after the boost revealed differences between GP coats at each GE dose, with V3042 GP packaged VRP inducing the highest level of SFCs at each dose tested (Figure 2). Only the ELISPOT responses detected in animals immunized with VRP packaged with the V3042 GP coat remained significant between different GE dosing groups (Figure 2). ELISA analysis of post-boost samples revealed a dose response effect between groups receiving different GE doses and some small but significant differences between GP coats largely in animals that received the highest VRP dose (Figure 3). The difference between the V3042 and TC-83 ELISA responses were mirrored at the 4.5×10^7^ GE and 4.5×10^5^ GE doses. The only other set of responses that remained significantly different between two different GE doses were V3014 and TC-83 in the 4.5×10^7^ GE and 4.5×10^3^ GE groups.

## Discussion

VRP have been shown to induce a broad array of immune responses to the foreign gene product expressed by the replicon, including cytotoxic T lymphocytes (CTL), lymphoproliferative responses and neutralizing antibodies. Moreover, VRP have been shown to confer protection in animal models against a variety of diseases that require humoral and/or cellular effector mechanisms for protection [13]–[22], [32]–[35].

One of the reasons that VRP vaccines are so potent in their ability to induce immune responses may relate to their ability to target dendritic cells (DC), which are the most potent antigen-presenting cells of the immune system. The lymphotropic nature of VEE virus has been known for many years, but it has only recently been appreciated that VEE virus specifically infects DC in the draining lymph nodes of mice, and VRP target the same cells [10]. VRP also infect human DC *in vitro* [12]. Recent data have demonstrated the specific usage of DC SIGN and L-SIGN as attachment receptors for alphaviruses and may mechanistically explain their DC-targeting phenotype. In addition to lymphoid specificity, recent studies suggest that the immunogenicity of alphavirus replicon vaccines may be also be influenced by the activation of the innate immune system, via ribonuclease L or a double-stranded-RNA-dependent protein kinase (PKR), that occurs in cells infected with an alphavirus or alphavirus replicon [36]–[40]. Thus, the DC targeting and high protein expression that stimulate the adaptive immune responses, along with signaling of the innate immune system by dsRNA replicative intermediates, combine to make the VEE replicon system an attractive vaccine vector.

Although VRP have demonstrated ability to induce robust immune responses only VRP packaged in a few different VEE GP coats have been tested in animals. VRP packaged with the V3014 GP have been examined the most [13]–[22], [32]–[35]. Other studies have included VRP packaged with V3000 GP: [10], [41], [42], V3010 GP: [10] and V3533 GP: [10]. Because the VRP doses used in these studies did not take particle to infectious unit ratios into consideration it is not possible to compare the immune responses demonstrated between these studies. As such, a direct comparison of the immunogenicity of VRP packaged with different VEE GP coats has not been described previously.

Data presented here suggest that large differences in Vero cell infectivity exist between VRP packaged with the V3000 GP and V3042 GP coats compared to V3014 GP and TC-83 GP coats. This is perhaps not unexpected for V3000 GP packaged particles compared to V3014 GP and TC-83 GP packaged particles because of the methods used to produce these respective attenuated viruses. The V3014 GP mutations were identified in an attenuated VEE virus with rapid binding and penetration characteristics on BHK cells [2], [43] and the TC-83 GP mutations were identified in an attenuated VEE virus after multiple cell culture passages [31], [44]. Both GP coats have key attenuating mutations that map to the E2 coding region and confer a strong heparan sulfate-binding phenotype [7]. The heparan sulfate-binding capacity of the V3014 GP and TC-83 GP may explain why VRP packaged with these coats demonstrate a low GE/IU ratio based on Vero cell infectivity. It is well accepted that tissue culture adaptation of VEE virus leads to selection of heparan sulfate binding mutations in the GP coat [7], but attenuating mutations in the GP coat of VEE viruses unrelated to heparan sulfate binding have been identified [5], [28]. An example of this is the V3042 virus. The GP coat of V3042 virus has a Phe→Ile amino acid change at position 81 of the E1 protein [6], [28] and this GP coat does not impart a strong heparan sulfate binding phenotype. The lower heparan sulfate binding capacity of VRP packaged with the V3042 GP coat (and V3000 GP coat) likely contributes to a GE/IU ratio, determined on Vero cells, that is 5–10 times that of V3014 GP and TC-83 GP coat packaged VRP. The weak heparan sulfate interaction is supported by the apparent increase in IU titer (and resultant reduction in GE/IU ratio) noted when VRP titration was carried out in low NaCl concentration buffers. It is possible that the lower ion concentration in the titration buffers may have allowed weak heparan sulfate charge associations with these GP coats that would normally not be strong enough to facilitate cell binding and VRP entry.

We have previously explored the use of the V3000 GP coat to package VRP and attempted to determine whether this coat could impart higher immunogenicity to particles when compared to that of the V3014 GP coat. Those preliminary results suggested that V3000 GP packaged VRP induced more robust immune responses in mice than did V3014 GP packaged VRP, but differences in the methods used to purify the two VRP made interpretation of the results difficult (Kamrud and Smith unpublished results). To eliminate differences in VRP purification, that may affect immunogenicity, a uniform production process was used for all VRP used in this study. In addition, particle to infectious unit ratios, as estimated by measuring GEs, were measured by RTqPCR to control for differences in particle number that may not be evident when VRP titer determined on Vero cells was the sole method used to assess potency.

The greater than 100-fold difference in GE/IU ratios between V3000 GP and V3014 GP packaged VRP may, in part, explain the preliminary results alluded to above. To assist in our analysis, only immune responses shown to be statistically different between GP coats that occurred in at least two of the tested GE dosing groups were considered. With this limitation, the majority of differences that were found between GP coats were seen only at the T-cell level by γIFN ELISPOT. Interestingly, VRP packaged with the V3000 GP coat induced the highest ELISPOT results after the prime while VRP packaged with the V3042 GP coat induced the highest ELISPOT results after the boost. It is unclear at this time why this was the case. The ELISPOT data suggest that induction of T-cell responses may be more sensitive to differences in VEE GP coat than induction of B-cell responses based on ELISA. Clearly, VRP induction of T-cell responses occurred rapidly as GAG-specific γIFN secreting cells could be detected 7 days after the prime while a GAG-specific ELISA response could not. It is possible that the nature of the GAG antigen is the basis for the low post prime humoral immune response noted; VRP packaged with the different GP coats that express an alternative antigen should be tested in the same manner to explore this further. Post-boost, the GAG-specific ELISA analysis showed a dose response effect independent of the GP coat used to package the VRP. Both the V3014 and V3042 GP VRP demonstrated significantly higher ELISA responses when compared to TC-83 GP VRP at two different GE dose levels. These data suggest that VRP packaged with V3014 or V3042 GP coats may be better at inducing humoral immune responses than TC-83 GP packaged particles but they are no different than V3000 GP coated particles. Similar studies have been conducted using either VEE or Sindbis (SIN) replicon vectors packaged in homologous or heterologous GP coats, respectively [41]. The results from that study indicated that the highest humoral and cellular immune responses detected in immunized animals correlated with the VEE non-structural genes used in the animals, not with the VEE or SIN GP coats used to package the VRP [41]. Our data suggest that some of the VEE GP coats examined here impart large differences in Vero cell infection capacity which results in a significant under estimation of the actual particles present in those VRP preparations. When animals are immunized with VRP packaged with different GP coats based on genome equivalents rather than Vero cell infectious units those differences are not mirrored in BALB/c mice based on the immune responses detected. That is, those particles not detected by Vero cell-based infection assay (IU) appear to be functional *in vivo* in BALB/c mice. Finally, these data suggest that caution should be taken when examining alternative GP coats used to package vector-based vaccines (such as adenovirus, adeno-associated virus, vesicular stomatitis virus and alphavirus-based vectors) as differences in apparent immunogenicity imparted by the GP coat may be an artifact of the method/cell type used to determine IU titer and immunization dose.

## Materials and Methods

### Cells and media

VRP production and titration were conducted using a certified Vero cell line derived from a master cell bank prepared from cells obtained from the World Health Organization. Vero cells were grown in Minimum Essential Medium (MEM: Invitrogen, Carlsbad, CA) medium containing 5% fetal bovine serum (FBS, HyClone, Logan, UT), for expansion before electroporation. After electroporation Vero cells were seeded into OptiPro (Invitrogen) serum-free medium with 2 mM glutamine.

### Construction of GAG replicon and GP helpers

The Du422 *gag* gene has been described previously [24]. The *gag* gene was cloned into an optimized replicon vector containing an Encephalomyocarditis virus (EMCV) IRES element and spacer sequence as described previously [9]. Site directed mutagenesis was carried out using a QuikChange® Site-Directed mutagenesis kit (Stratagene, La Jolla, CA) to introduce a G→A change at nucleotide position 3 (nt3A) at the 5′ end of the replicon RNA. The nucleotide 3 G→A mutation is a known VEE virus attenuating mutation [31] and was incorporated into the replicon to add an additional measure of safety to VRP generated with the V3000 GP coat. The nt3A, IRES-optimized, GAG replicon RNA was packaged with each of the different VEE GP coats analyzed in this study.

Helpers coding for the GP genes defined by the sequence of the VEE infectious clones V3000, V3014, V3042 and TC-83 were constructed. Construction of the V3014 GP helper, pHGP3014, has been described previously [8]. The V3000 GP gene was derived from the infectious clone of the wild type virulent Trinidad donkey strain of VEE [43]. The V3000 GP was cloned as a SpeI and SphI gene fragment into the V3014 GP helper plasmid digested with the same enzymes replacing the V3014 GP gene to generate the V3000 GP helper, pHGP3000. The TC-83 GP gene was digested from the full length infectious clone of VEE TC-83 virus [31] and cloned into a helper plasmid as described above to generate the TC-83 GP helper, pHGPTC-83. The V3042 GP helper was generated by introducing the E1-81 (Phe→Ile) mutation [28] into the pHGP3000 GP helper using site directed mutagenesis (Stratagene) as described above. The V3042 GP helper, pHGP3042, was sequenced to ensure that no errors were introduced during the mutagenesis reaction.

### RNA transcription, electroporation, VRP production and VRP titration

VRP were produced by co-electroporating Vero cells with replicon RNA combined with capsid and GP helper RNAs (sometimes referred to as the split helper system). The methods used to *in vitro* transcribe replicon, capsid and GP RNA and electroporate the RNAs into Vero cells have been described previously [9]. The infectious titer of VRP was determined by immunofluorescence assay (IFA) using goat anti-VEE nsP2 specific polyclonal antiserum as the primary antibody and donkey anti-goat Alexa Fluor 488 (Invitrogen) as the secondary antibody on methanol fixed Vero cells using a Nikon Eclipse TE300 fluorescence microscope. VRP were serially diluted in MEM (5% FBS), dilutions were inoculated onto Vero cells and analyzed 18 hr post infection as described above by IFA to determine an initial IU/ml titer. For VRP titers determined in low NaCl titration buffers, each VRP preparation was diluted to within two orders of magnitude of the original IU/ml titer determined in MEM (5% FBS). The VRP were then diluted 1:10 in one of the low NaCl titration buffers (20 mM sodium phosphate + 5.5 % sucrose, pH 7.0 and pH 7.4 or 20 mM Tris + 5.5 % sucrose, pH 7.4 and pH 8.0) followed by serial 2 fold dilutions in the respective low NaCl titration buffers. The VRP dilutions were inoculated onto Vero cells and analyzed as described above by IFA to determine IU/ml titer. The VRP were tested for the presence of contaminating replication competent VEE (RCV) using two blind passages on Vero cells as described previously [9].

### VRP purification

VRP were collected 18 hr post-electroporation, by removing the media and washing the cells with 0.4 M NaCl (salt wash). Some of the GP coats produced VRP that bound to Vero cells with less affinity than V3014 GP VRP, thereby resulting in a significant proportion of VRP in the media. In order to collect all of the VRP generated with each GP coat and to maintain a uniform purification method for all VRP the salt wash was combined with the media from electroporated cells and the pool concentrated 10 fold on a tangential flow filtration (TFF) system with 100,000 molecular weight cutoff Hydrosart membrane (Sartorius, Edgewood, NY). A 5 M NaCl /10 mM sodium phosphate pH 7.3 solution was added to the TFF retentate to produce a solution with a final sodium chloride concentration of 2 M. The solution was recirculated 5 minutes with the permeate closed and then the solution was concentrated to one half the original volume. The solution was diafiltered against 3 volumes of cold phosphate buffered saline containing 3 mM magnesium chloride. The permeate of the system was closed, 25,000 U of Benzonase (EM Science, NJ) was added and the system was allowed to recirculate for 5 min. The pump was stopped for 60 min and then restarted. A 5 M sodium chloride /10 mM sodium phosphate pH 7.3 solution was added to the TFF retentate to produce a solution with a final sodium chloride concentration of 2 M. The solution was recirculated 5 minutes with the permeate closed and then the solution was concentrated to one half the original volume. The solution was diafiltered against 3 volumes of cold 0.5 M sodium chloride/10 mM sodium phosphate pH 7.3. The TFF retentate was drained from the system, passed through a 0.2 µ filter and diluted 1:10 into 10 mM Tris buffer, pH 8.0. The diluted solution was loaded to a 3.5 mL Cellufine sulfate (Chisso, Japan) column at 200 cm/hr. The column was washed with 10 mM Tris, pH 8 followed by 10 mM sodium phosphate, pH 7.3. VRP were eluted with a step gradient to 1 M sodium chloride/10 mM phosphate, pH 7.3. Fractions were pooled based upon the A_280_ absorbance elution profile and the VRP titer in the pooled fractions was determined by IFA as described above.

### Quantitative reverse transcription PCR (RTqPCR) analysis of VRP

To determine the number of genome equivalents present in each different GP coated VRP, a standard one-step RT-qPCR protocol was performed on an Applied Biosystems 7500 Fast Real Time PCR System sequence detection system. Amplification was detected by means of a fluorogenic probe designed to anneal to a region of the nsP2 gene on the replicon between the two primers. A 5′ reporter dye (6-FAM) and a 3′ quencher dye (BHQ-1) were attached to the probe. Proximity of the reporter and quencher dyes resulted in the suppression of reporter fluorescence prior to amplification. Upon successful amplification of the target region, the 5′ exonuclease activity of DNA polymerase released the reporter dye from the hybridized probe, resulting in a fluorescent signal. Purified VEE replicon RNA was used to generate a standard curve in the assay, and the fluorescent signal of each VRP sample was measured over forty PCR cycles and compared to the fluorescent signal of the standards to determine genome equivalents.

### Vaccination of mice and sample collection

Female, 6–8 week old, BALB/c, mice (Charles River Laboratory, Kingston, NY) were immunized with VRP produced with the different GP coats based on genome equivalent (GE) titers. A summary of the GE titers, the respective Vero cell infectious titer and the number of animals immunized in each group for each dose are shown in Table 4. Mice were immunized at 0 and 3 weeks by subcutaneous (SC) injection into the rear footpad. GAG-specific antibody and T cell responses were monitored 1 week after each immunization. Blood was collected by retro-orbital bleeds for all groups. Eight (8) mice from each group were sacrificed 1 week after each immunization and splenocytes collected from individual animals for T-cell analysis.

### ELISA and ELISPOT analysis

For ELISA analysis, dilutions of sera from GAG-VRP vaccinated mice were made into PBS containing 1% BSA and 0.05% Tween-20 beginning with 1:40 followed by two fold dilutions out to 1:2560. Pre-bleed samples were diluted to only 1:40. ELISA plates (Nunc, Rochester, NY), which had been coated with HIV GAG antigen (0.25 µg/well) in carbonate/bicarbonate buffer (Sigma-Aldrich, St. Louis, MO) overnight at 4°C, were incubated with 200 µl/well of blocking buffer (PBS, 3% BSA) at 30°C for 1 hour. Blocked plates were washed three times with 200 µl of PBS. Fifty µl of diluted sera was added to the plates in duplicate and incubated at 30°C for one hour. Plates were washed three times with 200 µl PBS. 100 µl of alkaline phosphatase-conjugated anti-mouse polyvalent immunoglobulin's (IgG, IgA, IgM) (Sigma) diluted in blocking buffer (1:500) was added to each well. Plates with secondary antibody were incubated for 1 hour at room temperature and then washed three times with 200 µl PBS. Plates were developed using Fast™ p-Nitrophenyl phosphate tablet sets (Sigma) and reading at a wavelength of 405. An OD_405_ of 0.2 or greater was considered positive.

Splenocytes were isolated from individual animals and GAG specific gamma interferon ELISPOT assays were performed to determine the number of antigen-specific cytokine-secreting T cells. This procedure has been described previously [45].
